# The effects of occupational pollutants on the reproductive health of female informal street traders in Warwick junction, Durban, South Africa – a cross-sectional study

**DOI:** 10.1186/s12905-019-0854-7

**Published:** 2019-12-18

**Authors:** Sujatha Hariparsad, Rajen N. Naidoo

**Affiliations:** Discipline of Occupational and Environmental Health, University of KwaZulu-Natal, Durban, 4041 South Africa

**Keywords:** Informal traders, Reproductive health, Occupational pollutants, Biomass fuels, low birth weight

## Abstract

**Background:**

Informal street traders are exposed on a daily basis to traffic emissions and biomass fuel smoke containing a variety of pollutants. These exposures are likely to place the female traders at increased risk for adverse reproductive outcomes.

**Aim:**

The aim of this study was to compare the reproductive outcomes among street traders exposed to pollutants from their work-related activities and traders without such exposure.

**Methods:**

A cross-sectional study was conducted among 305 female traders selected from exposed and non-exposed areas within the Warwick Junction trading hub, located in Durban, South Africa. Validated reproductive questionnaires and clinical assessments were conducted on all participants. Adverse reproductive outcomes such as low birth weight, spontaneous abortions and infertility were assessed.

**Results:**

The mean age of the traders was 43.6 years (SD:12.1), mostly single (63%) and worked in trading hub for an average of 14 years. There were 876 pregnancies reported in the total sample. Traders pregnant while working in this location accounted for 120 pregnancies There was an increased risk of exposed traders having a low birth weight infant as compared to non-exposed traders (OR = 3.7; CI: 1.8, 7.6). Exposed traders were also almost 3 times more likely to be infertile as compared to non-exposed traders (OR = 2.6; CI: 1.6, 4.3).

**Conclusions:**

This study has demonstrated that female street traders working within a trading hub in KwaZulu-Natal, South Africa; exposed to occupational pollutants show an association with developing infertility and low birth weight infants. Recommendations to mitigate adverse exposures have been suggested which include short term safety interventions and better cooking practices and future long term policy interventions.

## Background

Adverse reproductive outcomes have long been a cause of concern worldwide. The World Health Organisation (WHO) reported a global estimate of 130 million births annually, 303,000 resulted in maternal mortality. Still births and neonatal deaths account for 2.6 million and 2.7 million respectively [1]. A vast majority of these adverse outcomes occur in developing countries. In 2009, South Africa reported having at least 12.3% of births being classified as low birth weight with India leading at 27.6% low birth weight babies [2].

Nearly 80% of neonatal deaths are attributed to low birth weights, premature delivery, asphyxia and birth trauma.

The inability for a woman to have a successful healthy birth has been classified as an adverse reproductive outcome [1]. The Demographic Health Survey conducted in 2000 by WHO, determined that at least 186 million women in developing countries were unable to conceive [3].

There is growing scientific literature and evidence to suggest that the workplace and environmental exposures may have a relationship with adverse reproductive outcomes. An increased risk of adverse birth outcomes was found in a cohort of employed Finnish women [4]. Risks of low birth weight (OR = 3.7; CI 1.2–11.6) and small for gestational age (OR = 1.5; CI 0.7–3.2) were higher when compared to unemployed women [4]. The study also concluded that women working within the occupational group of mining, construction and factories had increased risk of delivering low birth weight infants (OR = 3.7; CI 1.2–11.6).

Due to the burden of poverty and unemployment many discouraged job seekers enter the informal sector [5]. In 2013, South Africa reported an estimated 1.4 million persons involved in the informal sector [6]. The quarterly labour force survey released in June 2017 indicated a 10% increase in employment within the informal sector as compared to 2016 [7]. The trading industry accounts for 54.4% of the informal businesses within the sector. Women are the driving force within this industry with 52.1% women in trading alone [6].

In Kwazulu-Natal, the proportion of working age population involved in informal businesses is 4.7% with Limpopo rated as the highest at 6.3% of individuals involved in informal businesses [6]. Informal traders in KwaZulu-Natal are predominantly women (58%) who are also predominantly involved with trading food [5].

Informal street vendors are at risk of cumulative exposure to multiple occupational hazards due to their non-specific job activities [8]. Maternal health has been shown to be negatively affected by perennial physical exhaustion, physical abuse and inherent stress that arises from street trading [9].

Environmental studies describe the adverse effects of air pollutants, such as particulate matter (PM), carbon monoxide (CO), sulphur dioxide (SO2) and nitrogen oxides on reproductive health of women [10]. Increased levels of PM10 and PM2.5 were found to have a causal association with low birth weight deliveries, pre-term labour and small for gestational age neonates [10–12]. It was noted that with a 20 μg/m^3^ difference in PM10 levels in the third trimester there was a decrease of birth weight by 21,7 g (95%CI, 1.1–42.2 g).

Preparing and cooking food is a key trading activity among informal street traders due to the high income generated from sales [8, 13].. To ensure low costs, street traders use cheaper, more accessible forms of fuel source, such as biomass fuels [9, 14, 15].

Garbage burning practices and use of biomass fuels were investigated in 592 mothers and new-born infants in Accra [15]. Increased risk of adverse reproductive outcomes were noted in the women using charcoal (RR 2.4; CI 1.3, 4.4) and garbage (RR = 2.9; CI 1.1, 7.9) as fuel sources. A cohort of 19,270 singleton birth weights were analysed based on the types of fuels used by the mothers during pregnancy [16]. The fuel types were categorised according to high pollution fuels (biomass fuels, charcoal, and kerosene) and low pollution fuels (electricity, liquid petroleum gas). The use of high pollution fuels was significantly associated with low infant birth weights (OR = 1.4; CI 1.3, 1.6).

This study conducted among female street traders in Warwick Junction (WWJ) trading hub, Durban, South Africa aimed to describe the association between reproductive health of these traders and their workplace exposures.

## Methods

### Study design and setting

This cross-sectional study was conducted among female street traders in Warwick Junction between the months of March and May 2015.

Traders were sampled from the various markets within the trading hub; namely mealie (corn) cookers, bovine head market, traditional herb market, clothing market, fresh produce market and roadside vendors. The registry of traders and registered stall holders were obtained from an informal census conducted by a local non-governmental organisation, Asiye eTafuleni (AeT) in 2013.

Only female traders above 18 years of age who were currently working in WWJ for any given time period were included in the study. Participants were included into the study following informed consent.

Information gathered during interviews were considered confidential. All data captured and analysed was reported as group data and results. Individual data was not disclosed. Participants were reviewed following questionnaires and biological monitoring by the principal researcher, a medical practitioner. Results and information gathered that warranted immediate medical intervention were duly attended to via referral to the relevant disciplines within the public health sector.

### Population and sample selection

The total number of female traders sampled in the Warwick Junction trading hub was 305, with 25% in traditional herb market and 59% in cooking activities (bovine head market and mealie cookers).

The traders were divided into exposed (*n* = 168 (55%)) and non-exposed (*n* = 137 (45%)) groups, based on their occupational exposures. The exposed group consisted of traders from the bovine head market, mealie cookers and traders in the traditional medicine market, all exposed to either biomass fuels used in cooking, or dusts, while the non-exposed were from the clothing market, fresh produce market and roadside vendors.

Due to the limited number of traders working solely in the cooking processes, the full complement of mealie cookers (*n* = 50) were recruited and all the bovine market traders available during the sampling period were recruited (*n* = 43 (72% of the bovine traders)).

The remaining traders were selected randomly from registry of traders present at the market. The lists of traders from the various markets were provided to the research team during the sampling period. From these lists an estimated 10–15% of the traders were randomly selected to participate in the study.

### Participant interviews

Demographic data, relevant medical history and data on current and previous employment were recorded via a questionnaire.

Reproductive health was assessed via previously validated questionnaire used in the National Birth Defects Prevention Study conducted in the United States [17]. Participants were asked to report on five previous pregnancies (where applicable) and their most recent pregnancy. Detailed questions were asked on characteristics of birth based on recall of the participants (foetal weight, sex, year, outcome), doctor diagnosed conditions during the respective pregnancies (gestational diabetes, eclampsia, hyper-emesis gravidarum), use of over the counter medication and traditional medicines and history of smoking, alcohol and drug use during pregnancy. Infertility was assessed via questions on difficulties experienced during conceiving, being diagnosed as infertile by a healthcare practitioner, treatment taken for infertility and current and past history of relationships with other partners (duration of relationship, duration of un-protected sex, if partner had conceived previously).

### Statistical analyses

Data was coded and captured using double entry into Microsoft Excel. Statistical analysis was conducted via Stata IC version 13.1 software for Windows.

Dependent variables were reproductive outcomes: (1) number of pregnancies, (2) spontaneous abortions, (3) low birth weight and (4) doctor diagnosed infertility.

Low birth weight was defined as weight at birth of less than 2500 g [1]. Spontaneous abortions/ miscarriages was defined as the premature loss of a foetus up to 23 weeks of gestation [18]. Infertility was defined as the inability of a sexually active couple to achieve pregnancy in one year [19].

Independent variables were defined as exposure to trading in WWJ (calculated in years) and trading area.

Univariate analysis, frequency tables and descriptive statistics was used to describe means, range and standard deviations. Means and standard deviations were compared for numerical variables using student’s t test. Categorical variables were analysed using Pearson’s chi square test to determine measures of association between respiratory symptoms and exposure.

Multivariate regression modelling (linear and logistic) was used to determine the association between adverse reproductive outcomes (dependent variables) and independent exposure variables. The multivariate analysis adjusted for covariates, including age, diagnosed chronic conditions, biomass fuel use at home and type of trading activity. Models were designed for reproductive outcomes among traders who were pregnant while working at WWJ. These models were adjusted for age, doctor diagnosed chronic condition, years working in WWJ and biomass fuel use at home. The statistical level of significance was maintained as *p* < 0.05 with 95% confidence intervals.

## Results

The traders were middle aged (mean: 43.6 SD:12.1) and mostly single (63%). The majority of the traders reported completing secondary schooling or Grade 12 [174(57%)]. (Table 1).

**Table 1:** Demographic data (*n* = 305)

Characteristics	N (%)
Age (mean) (years) (SD)	43.6 (12.1)
Marital Status	
Single	192 (63)
Married	75 (24)
Education Level	
Never attended school	51 (17)
Completed primary school	80 (26)
Completed secondary school	174 (57)
Self-reported doctor diagnosed chronic conditions	153 (50)
HIV + ve	67 (22)
TB (past history)	12 (4)
Hypertension	46 (15)
Non-insulin dependent diabetes mellitus (NIDDM)	28 (9)
Tobacco use (snuff)	3 (1)
Years working in WWJ* (mean)(years) (SD)	14 (10.03)
Trading Area	
Mealie cookers	50 (16)
Traditional herb market	75 (25)
Bovine head market	43 (14)
Early morning market	34 (11)
Clothing market	81 (27)
Roadside food vendors	14 (5)
Other	8 (3)

There were 167 (55%) of the participants in the exposed group. These were the mealie cookers [*n* = 50 (16%)], bovine head market [*n* = 43 (14%)] and the traditional herb market [*n* = 75 (25%)]. There were 93 (30%) traders involved solely in cooking practices (the bovine head market and mealie cookers) within the sample. The remaining traders were considered non-exposed and belonged primarily to the clothing markets [*n* = 81 (27%)], fresh produce market [*n* = 34 (11%)], roadside vendors [*n* = 14 (5%)] and other traders in sales of goods [n = 8 (3%)]. The participants on average were working in WWJ for 14 years with a small minority (5%) working for greater than 30 years in the market. There were no statistical significant differences in demographic profiles between the exposed and non-exposed groups.

Traders in primarily cooking practices used fuel sources ranging from treated wood, coal, paraffin and combination of fuels. A small percentage (5%) of traders used combustible materials such as plastics, paper and garbage refuse as fuel sources. The remaining traders used predominantly treated wood and coal as a primary source of fuel.

A vast majority of traders [*n* = 289 (95%)] reported being pregnant at least once. The overwhelming majority (*n* = 261 (90%)) were multiparous. (Table 2). The total number of pregnancies among the traders was 876, with 808 live infants with a male: female ratio of 0.9:1. Adverse reproductive outcomes, ranged from a low prevalence of ectopic pregnancies (0.3%) through to the reporting of low birth weight (7%). Within the total sample, exposed traders had increased parity as compared to non-exposed (*p* = 0.004). Doctor diagnosed infertility also accounted for 6% (*n* = 17) of the total number of participants.

**Table 2:** Reproductive Health of Traders (N = 305)

Reproductive Characteristics	N (%)
Gravidity (pregnant before)	289 (95)
Parity (n = 289)*	
Primigravidity	28 (9)
Multiparous	200 (69)
Grand multiparity	61 (21)
Reproductive Outcomes	
Total number of pregnancies	876
Live infants	808
Sex of infants	
Males	394 (49)
Females	414 (51)
Spontaneous abortions	23 (3)
Ectopic pregnancy	3 (0.3)
Low birth weight	58 (7)
Doctor diagnosed infertility	17 (6)

*Primigravidity – woman who has been pregnant once only

Multiparous – women who has had 2–4 pregnancies.

Grand multiparity – woman with 5 or more completed pregnancies reaching gestational viability.

Traders becoming pregnant while working in WWJ was analysed as a subset of the data (Table 3). There were 71 (59%) traders working in exposed areas while pregnant. There was a total of 120 pregnancies with 111 live infants born while working in WWJ.

**Table 3:** Reproductive Characteristics of Traders pregnant whilst working at WWJ (*N* = 120)

Reproductive Characteristics	N (%)
No. of pregnancies	120
Live children	111 (93)
Parity (*n* = 120)	
Primigravida	7 (6)
Multiparous	78 (65)
Grand multiparity	35 (29)
Spontaneous abortions	9 (8)
Low birth weight	35 (29)

The majority of the pregnancies during work at WWJ, were among traders in the traditional herb market [*n* = 34(28%)]. There was a collective high prevalence of low birth weight (34%) and spontaneous abortions (10%) among traders in the exposed group compared to non-exposed.

Infertility (*p* = 0.002) and low birth weight (*p* = 0.000) were significantly higher in exposed traders compared to those without exposure. (Table 4).

**Table 4:** Adverse reproductive outcomes in traders pregnant whilst in WWJ stratified according to trading areas

Reproductive outcome	N (%) (n = 120)	Exposed areas (*n* = 71)			Non-exposed areas (*n* = 49)		
		*Mealie cookers (n = 19)*	*Bovine head market (n = 18)*	*Traditional herb market (n = 34)*	*Clothing market (n = 23)*	*Fresh produce + roadside vendors (n = 24)*	*Other (n = 2)*
Spontaneous abortions	9 (8)	1 (2)	2 (5)	4 (5)	2 (3)	0	0
Low birth weight*	35 (29)	9 (18)	4 (9)	11 (15)	6 (7)	3 (6)	2 (25)
Infertility(n = 305)**	17 (6)	4 (8)	1 (2)	5 (6)	4 (5)	2 (4)	1 (13)

*p = 0.000

**p = 0.002

Regression analysis showed that an increased significant exposure related risk existed for infertility (adjusted OR = 2.6; CI 1.6, 4.3) (Table 5) Traders who fell pregnant while working in WWJ were also at a high risk of delivering infants of low birth weight as compared to traders who were not exposed during pregnancy [adjusted OR = 3.7 (1.8,7.6)]. Regression models adjusted for smoking history, years working in WWJ, history of chronic conditions and use of biomass fuel at home. Alcohol was not considered in the models as none of the traders reported consuming alcohol.

**Table 5:** Logistic regression adverse reproductive outcomes amongst traders pregnant whilst at WWJ stratified by exposed and non-exposed

Reproductive Outcomes whilst @WWJ	Exposed (*n* = 120)		Non-exposed (*n* = 185)	
	Unadjusted OR (95%CI)	Adjusted OR (95%CI)	Unadjusted OR (95%CI)	Adjusted OR (95%CI)
Spontaneous abortion	0.9 (0.4, 2,4)	1.1 (0.4, 3.2)	1.0 (0.4, 2.4)	0.9 (0.3, 2.6)
Low birth weight*	2.9 (1.6, 5.2)	3.7 (1.8, 7.6)	0.3 (0.2, 0.6)	0.3 (0.1, 0.5)
Infertility*	2.4 (1.5, 3.9)	2.6 (1.6, 4.3)	0.6 (0.5, 0.8)	0.6 (0.5, 0.8)

*statistically significant CI

** Models were adjusted for smoking, years working in WWJ, chronic conditions and use of biomass fuel at home

## Discussion

This study among informal street traders provides additional evidence of increased exposure related risk for adverse reproductive outcomes [20]. The findings in this study show statistically significant risks associated with trading while pregnant and delivery of low birth weight infants. Risk estimate for low birth weight was reported as 3.7 (CI 1.8, 7.6) in this study.

This is higher than reported in the literature. In Accra, Ghana a cross-sectional study on 105 female street vendors indicated an association between street vending and adverse reproductive outcomes [8]. The average birth weight of babies born to street vendors were 177 g (CI -324, − 31) lighter than babies born to women employed in administrative, seamstress and professional jobs. This indicated an increased risk of delivery of low birth weight infants (RR = 1.8; CI 1.0, 3.1) amongst the street vendors.

Fuel use amongst pregnant women and the associations with adverse reproductive outcomes have been extensively researched. A fixed effect meta-analyses conducted by Pope et al., described the relationship between use of biomass fuels and birth weight. Use of solid biomass fuels was significantly related to low birth weights (OR = 1.4; CI 1.3, 1.5) and a reduced mean birth weight (− 95.6 g; CI -68.5, − 124.7, 21]. Findings in the analysis of the 2005–6 India Demographic Health Study also showed the highest effect on birth weight to be type of fuels used. Use of biomass fuels accounted for an increased risk of low birth weight (OR = 1.4; CI 1.3, 1.6, 16].

Cooking fuel choices vary amongst informal traders throughout the world. A cross-sectional study of 592 mothers in Accra, Ghana in 2010 demonstrated that women burning garbage as a cooking fuel were exposed to carbon monoxide (CO) and particulate matter (PM) which was emitted in the smoke. Garbage burning was associated with an increase in the risk of low birth weights (RR = 2.95; 95% CI 1.10; 7.92, 15].

Traders in Warwick Junction make use of domestic waste by products as an easily accessible cheap fuel source [21]. Treated wood was also a primary source of fuel amongst the traders. Wood use has been shown to impact adversely on pregnancy outcomes as reported in central east India [22]. In this study the researchers studied a cohort of 1744 women enrolled at delivery. The study found that compared to gas, women using wood were significantly more at risk of delivering preterm babies (OR = 3.11; CI 2.12;4.59) [22].

A study conducted among 422 female street traders in Johannesburg South Africa, found high prevalence of infertility (22%) and miscarriages (13.7%). Another 13.5% of the women reported experiencing difficulties in conceiving during their child bearing years [13]. Similarly to this study conducted by Pick et al., spontaneous abortions also accounted for a small percentage (8%) of the pregnancies of women working at WWJ.

Infertility was also identified as a significant risk associated amongst exposed street traders in this study (adjusted OR = 2.6; CI 1.6, 4.3). South Africa has been leading the trend of increasing infertility within sub-Saharan Africa, with a national fertility rate of 2.8 [23]. In the Johannesburg study 22% of these women reported being infertile [13].

We were unable to assess further history of previous pregnancies such as; frequency of antenatal visits, clinical assessments during the pregnancies and other psychosocial factors (stress, nutrition status,etc) that could affect the outcome of the pregnancy. Information bias might have also existed amongst traders who had to recall information on their previous pregnancies which could not be objectively verified.

A further limitation is that we were unable to conduct personal exposure sampling while the women were pregnant. However since trading activities seldom change and traders are not in fixed isolated trading areas, we adjusted for crude exposure readings which were based on years working in WWJ.

## Conclusion

Our findings indicate a risk of adverse reproductive outcomes in the informal sector in South Africa. Access to healthcare and education of traders on reproductive and maternal health needs to be a priority to assist in reducing incidence of adverse reproductive outcomes in this vulnerable group of workers. Short term interventions need to be assessed in this working environment. Control measures; such as engineering, administrative and use of personal protective equipment have been previously implemented such as; designing of separate stalls, chimneys and areas of natural ventilation to mitigate exposure to smoke emissions and dust. Informal street traders form an integral component of the economy and should therefore be accorded inclusion into labour legislation and national policy development.

## Implications for practice and policy

According to statistics released by the ILO, approximately 2.5 billion people, or half of the global workforce are employed within the informal economy [24]. Informal employment contributes to at least 66% within sub-Saharan Africa with 33% employment in South Africa.

The primary concern therefore remains the lack of legislation and inadequate health and safety control measures within this rapidly growing economy. Formal sector and informal sector are chiefly viewed as being separate entities however in most developing economies these two sectors overlap [25].

A notable step has been taken by the ILO to address the challenges of the informal economy by adopting Recommendation No.204 in 2015 which seeks to address the Transition from Informal Economy to the Formal Economy [24]. However in struggling economies with high levels of poverty and unemployment the objectives described in this Recommendation seem idealistic and unattainable.

The relevant stake holders; governmental and non-governmental need to engage and embark on planning and developing new policies and interventions to be implemented within this sector to assist in reducing the growing burden of disease by ensuring safer working practices.

Accessibility of healthcare poses a challenge to these informal traders. In an attempt to mitigate this, it is recommended that local government institute a mobile clinic program within the trading hub specifically for traders. This intervention will assist traders to utilize healthcare facilities whilst at work. By providing this service the burden of diseases prevailing in this community might be minimized. Early diagnosis and prevention of diseases will be encouraged by the implementation of this facility.

## Data Availability

The data is readily available from the corresponding author on reasonable request.

## Abbreviations

BMF: Biomass fuels
ILO: International Labour Organisation
WHO: World Health Organisation
WWJ: Warwick Junction
